# Communication with patients with limited prognosis—an integrative mixed-methods evaluation study

**DOI:** 10.1007/s00520-022-07474-9

**Published:** 2022-12-22

**Authors:** Anja Siegle, Laura Unsöld, Nicole Deis, Katja Krug, Jasmin Bossert, Johannes Krisam, Corinna Jung, Jana Jünger, Michel Wensing, Michael Thomas, Matthias Villalobos

**Affiliations:** 1 Department of Thoracic Oncology, University Hospital Heidelberg, Thoracic Clinic, Translational Lung Research Center Heidelberg (TLRC-H), German Center for Lung Research (DZL), Heidelberg, Germany; 2 Department of General Practice and Health Services Research, University Hospital Heidelberg, Heidelberg, Germany; 3 Institute of Medical Biometry and Informatics, University Hospital Heidelberg, Heidelberg, Germany; 4 Present Address: Medical School Berlin, Calandrellistr. 1-9, 12247, Berlin, Germany; 5 Institute for Communication and Assessment Research, Medical Faculty, University Heidelberg, Heidelberg, Germany

**Keywords:** Lung cancer, Terminal illness, Mixed methods, Communication, Prognosis, Palliative medicine

## Abstract

**Purpose:**

Oncological societies advocate the continuity of care, specialized communication, and early integration of palliative care. To comply with these recommendations, an interprofessional, longitudinally-structured communication concept, the Milestone Communication Approach (MCA), was previously developed, implemented, and evaluated. Our research question is: what are possible explanations from the patient perspective for prognosis and advance care planning being rarely a topic and for finding no differences between MCA and control groups concerning distress, quality of life, and mood?

**Methods:**

A pragmatic epistemological stance guided the study. A mixed-methods design was chosen including a pragmatic randomized trial (*n* = 171), qualitative interviews with patients (*n* = 13) and caregivers (*n* = 12), and a content analysis (133 milestone conversations, 54 follow-up calls). Data analysis involved the pillar integration process.

**Results:**

Two pillar themes emerged: 1 “approaching prognosis and advance care planning”; 2 “living with a life-threatening illness”. Information on prognosis seemed to be offered, but patients’ reactions were diverse. Some patients have to deal with having advanced lung cancer while nonetheless feeling healthy and seem not to be ready for prognostic information. All patients seemed to struggle to preserve their quality of life and keep distress under control.

**Conclusion:**

Attending to patients’ questions, worries and needs early in a disease trajectory seems key to helping patients adjust to living with lung cancer. If necessary clinicians should name their predicament: having to inform about prognosis versus respecting the patients wish to avoid it. Research should support better understanding of patients not wishing for prognostic information to successfully improve communication strategies.

**Trial registration:**

Registration: German Clinical Trial Register No. DRKS00013649, registration date 12/22/2017, (https://www.drks.de/drks_web/navigate.do?navigationId=trial.HTML&TRIAL_ID=DRKS00013649) and No. DRKS00013469, registration date 12/22/2017, (https://www.drks.de/drks_web/navigate.do?navigationId=trial.HTML&TRIAL_ID=DRKS00013469).

## Introduction

### *Background*

Lung cancer is one of the most frequent cancers in Europe and the most common cause of cancer-related death [1]. Worldwide the 5-year net survival rate is 10–20% [2], with most patients being diagnosed at an advanced stage of the disease. Despite advances in therapies for metastatic lung cancer, the disease remains incurable and the prognosis is limited for most patients [3].

Communication with lung cancer patients with limited prognosis comprises a series of breaking bad news along the disease trajectory: diagnosis, tumor progression, transition to best supportive care. Most importantly, the quality of care for these patients depends on the communication skills of their healthcare professionals [4]. Patient-centered communication includes comprehensive, intelligible, and truthful information. Patients have different information needs, different levels of resilience, and different coping strategies, and physicians need to tailor the amount and the content of information given to their patients [5].

Studies on physician–patient consultations show that patients seem to have difficulties in processing and recalling information concerning their disease and prognosis, especially if the treatment intention is palliative and not curative [6, 7]. From the patient perspective, physicians are responsible for initiating information on prognosis and advance care planning [8]. Furthermore, when mentioning prognostic information, physicians tend towards disease-orientated communication, focusing on tumor-specific therapy [6]. Patients with lung cancer seem to underestimate the extent of the disease and overestimate the cure possibilities [6], and their prognostic awareness swings to varying degrees [9]. Numerous high-quality studies demonstrate the inability of Advance Care Planning (ACP) to achieve its desired outcomes. There is supposed to be a gap between hypothetical assumptions and the decision-making process in clinical practice [10] which needs further investigation. Numerous communication interventions for breaking bad news and serious illness conversations have been developed and show positive outcomes for patients [11–13]. But communication interventions often fail to translate into meaningful patient care outcomes in routine clinical practice [14]. One possible reason is inadequate tailoring and implementation of the intervention within the given setting [15]. Therefore, projects must incorporate an implementation phase and implementation outcomes.

Before 2018, most studies on communication interventions used either a quantitative or qualitative approach to study feasibility and effects. Therefore, we chose qualitative and quantitative approaches within a mixed-methods design to gain an extensive understanding of what works and why it works [16]. Mixed methods allow data to be corroborated and conclusions to be derived from diverse perspectives [17]. They help in integrating results on unforeseen questions that arise during analysis. The data integration of mixed methods generates insights into, e.g., identifying subgroup characteristics, demonstrating parallels between scaled scores and behavioral categories, and showing patterns of relationships [18].

A milestone communication approach (MCA) to address communication needs has been developed, implemented, and evaluated using mixed methods. Some MCA results were analyzed separately and have been published [19–21]. The results of the pragmatic randomized controlled trial showed significantly fewer health system and information needs (SCNS-34-SF) in the MCA group compared to the control group (MCA: *M* = 33.4, SD = 27.5; standard care: *M* = 43.1, SD = 29.9, *p* = 0.033; effect size: Cohen’s *d* =  − 0.0.37) [22]. Other results raised questions in interpretation: the quantitative content analysis of patient records showed that prognosis and advance care planning were documented in fewer than 20–30% of the patient records [21] and the randomized controlled trial did not show differences between the MCA and control group for the secondary outcomes: distress, quality of life, and mood [19]. Data integration of the quantitative and qualitative results helped in gaining an insight in the answers to the following questions:

- What are possible explanations from the patient perspective for prognosis and advance care planning being a topic in fewer than 20–30% of patient records?
- What are possible explanations from the patient perspective for finding no differences between MCA and control groups concerning distress, quality of life, and mood (anxiety/depression)?

## Methods

### Milestones communication approach (MCA)—a complex intervention

MCA offers planned and structured milestone conversations along the disease trajectory involving the patient, family caregiver, nurse navigator, and physician. Milestone conversations take place during the following phases: (1) diagnosis, (2) tumor remission under treatment, (3) tumor progression, and (4) transition to best supportive care. All MCA intervention components can be seen in Table 1. MCA’s goal is (a) to respond to the communication needs of patients and caregivers, (b) to enhance continuity of care, (c) to support individual quality of life of patients and their caregivers, (d) to cultivate shared decision-making including end-of-life decisions, and (e) to promote communication proficiency and team processes of the interprofessional oncology team [23].

**Table 1:** MCA components and materials

MCA component	MCA materials
Interprofessional communication training (four sessions (6–8 h each) with 3 to 4 weeks in between)	- Milestone conversation (MC) manual for physicians and nurses: detailed description of the content of planned, structured nurse-physician–patient consultations including family caregivers (with rationale, objectives, definitions, and procedures) - Memory cards for physicians and nurses: a pocket-sized overview of essentials of the MCA conversation manual - Training materials: description of communication techniques and exercises for all four training sessions - Training observation checklist: list of essential components trainers use to evaluate MCA exercises during the training and coaching
Milestone conversation (in four phases over the disease trajectory)	- Milestone conversation file (shared electronic file for all members of the professional team) with the content of the consultations and reactions of patients and caregivers - Brochure for patients: managing lung cancer symptoms (developed by the Roy Castle Lung Cancer Foundation), translated into German, adapted to the German health care system
Follow-up session (1 week after each milestone conversation and then monthly)	- Follow-up sessions manual for nurses: detailed description of the content of follow-up phone calls or meetings for nurses - Follow-up chart with the content of follow-up sessions and the stated needs and reactions of patients and/or caregivers - Integrated palliative outcome scale (IPOS): instrument to measure palliative care needs of patients in relation to symptoms but also extending to information needs, practical concerns, anxiety or low mood, family anxieties, and overall feeling of being at peace

The milestone conversations contain breaking bad news, planning advanced cancer care, assessing prognostic awareness, and enhancing communication competencies and treatment choices [5, 9, 22]. A nurse navigator with palliative care experience contacts the patients 1 week after each milestone conversation and then at least monthly. During the follow-up, the nurse navigator answers questions, assesses palliative needs using the Integrated Palliative Outcome Scale (IPOS) [24] and consults or refers depending on the physical, psychosocial, and health system problems stated by the patients. The Intervention development and the intervention have been described in more detail [23, 25].

### Design and setting

The MCA study involves a variety of procedures and leads to a complex study design. The goal of the overall study was to evaluate MCA training, implementation context and outcome, patient outcomes, and effects on interprofessional collaboration. To capture the perspectives of those involved (patient, family caregiver, and healthcare professional) and evaluate different intervention components (milestone conversation, follow-up, training, and implementation) at different stages (development, implementation, and evaluation), a multiphase mixed-methods design was chosen [23]. For the present study, the data to answer the research questions were derived from three selected quantitative and qualitative methods: a pragmatic randomized controlled trial, a semi-structured interview study, and a content analysis of patient records.

A pragmatic epistemological stance guided the study. In pragmatism, the focus is on the consequences of research. The research is oriented towards what works in real-world practice [16]. This report follows the criteria of the Good Reporting of A Mixed Methods Study [26].

The study was conducted at the Department of Thoracic Oncology, University Hospital Heidelberg, a comprehensive cancer center and one of the largest lung cancer centers in Germany.

### Recruitment and sampling

From May 2018 to January 2020, a study nurse invited patients in the Department for Outpatient Oncology Services to participate. The recruited participants served for quantitative and qualitative data collection. The inclusion criteria for all participants were 18 years or older, recently diagnosed with metastatic lung cancer (stage IV) and having an acceptable command of German. All participants gave written informed consent. The study received an ethical clearing by the responsible Ethics Committee for Phase 1 (No. S-139/2017 on May 30th 2017) and Phase 2 and 3 (No. S- 561/2017 on 29th November 2017).

### Data collection

Raw data of the randomized controlled trial, the content analysis of patient records and the qualitative interviews were provided within the study group (KK and JB).

#### Randomized controlled trial

The primary outcome of the study was the dimension Health System and Information Needs of the Short Form Supportive Care Needs Survey (SCNS-SF34-G) measured 3 months after inclusion in the study. Compared were patients receiving MCA and patients receiving standard oncological care. Secondary outcomes included other physical and psychological supportive care needs (SCNS-SF34-G), quality of life (Schedule for the Evaluation of Individual Quality of Life (SEIQoL) and the Functional Assessment of Chronic Illness Therapy – Lung module (FACT-L)), distress (Distress-Thermometer (DT)), and depression and anxiety (Patient Health Questionnaire (PHQ-4)). Participating patients were asked to fill in questionnaires at the time of inclusion (baseline, t0), after 3 (t1), 6 (t2), and 9 months (t3). Descriptive raw data sheets and tables were provided.

#### Content analysis of patient records

Partly standardized routine records were used for MCs and follow-up documentation and collected by the nurse navigators of MCA. All topics addressed by patients, physicians, and nurses were documented in free text. For analysis, contents of the patient record were analyzed using qualitatively developed checklists for MCs and follow-up calls. Written descriptive data was provided.

#### Semi-structured interviews

The face-to-face interviews were conducted additionally to a regular appointment at the clinic, in a quiet room at the outpatient department. A semi-structured interview guide was developed, tested, and used for patients and caregivers. The interview guide comprised open-ended questions to elicit information on experiences with the MCA. All interviews were recorded digitally and transcribed verbatim. The transcripts were compared with the digital recordings to correct any inaccuracies. All interview transcripts were provided. Table 2 shows the aim, dimension, data source, data collection, and collection period of each method.

**Table 2:** Quantitative and qualitative methods used for data integration

	Pragmatic randomized controlled trial [19]	Semi-structured interviews [20]	Content analysis of patient records [21]
Method	Quantitative	Qualitative	Quantitative
Aim	Evaluation of patient outcomes	Evaluation of patient outcomes	Evaluation of implementation fidelity
Dimension	SCNS-34 SF-dimension health system and information needs SCNS-34- SF–dimensions (psychological, physical and daily living, patient care and support), distress (distress-thermometer), quality of life (SeiQol, Fact-L), anxiety and depression (PHQ4)	Semi-structured interview guide: experiences with MCA, interprofessional tandem, conversation content, decision making, caregiver involvement	Self-developed fidelity checklist on six general topics (therapy, patient preferences, physical condition, psychological condition, organization, complementary medicine)
Inclusion criteria	Newly diagnosed stage IV lung cancer, limited prognosis (< 12 months median), sufficient command of the German language, at least 18 years old	Newly diagnosed stage IV lung cancer, limited prognosis (< 12 months median), sufficient command of the German language, at least 18 years old	All documents of the collection period were evaluated
Data source	Patients and caregivers	Patients and caregivers	Patient records
Data collection	Patients randomly assigned to MCA intervention (structured MCA conversation including nurse navigator follow-up contact) or standard oncological care. Randomization remained pragmatic because trained physicians were treating patients of both groups. Patients (*n* = 171) filled in questionnaires at baseline (t0), after three (t1), six (t2), and nine (t3) months	Face-to-face interviews with patients (*n* = 13) and caregivers (*n* = 12) of the MCA intervention group were conducted, digitally recorded, and transcribed verbatim	Routine patient records (133 milestone conversations, 54 follow-up calls) were collected by three MCA nurse navigators. In two observed periods, all patient records on MCA conversations and follow-ups were included in the analysis
Collection period	05/2018–04/2020	09/2018–04/2019	t1: 01/2018–05/2018 t2: 09/2018–10/2018
Previous data analysis	All measures were analyzed descriptively. Differences between groups were analyzed for the intention-to-treat population using linear models (baseline (t0) value as the independent variable)	Qualitative content analysis according to Mayring: summarizing the content, deductive line-by-line coding	For each record, incidences of the checklist were entered into a data matrix and descriptively analyzed
Data used for the Pillar integration process	Descriptive values	Transcripts were coded inductively according to Braun and Clarke [27]	Descriptive values

Abbreviations: *MCA* milestone communication approach, *SCNS-34 SF* supportive care needs survey—34-short form, *SeiQol* schedule for evaluation of individual quality of life, *FACT-L* functional assessment of cancer therapy—lung, *PHQ-4* patient health questionnaire, *UWE-IP* University of the West of England Interprofessional Questionnaire

### Data analysis

In mixed-methods research, data integration can be understood as an approach that merges, connects, or embeds qualitative and quantitative procedures [18]. Data integration was performed by AS (first author), a health care researcher and nurse, together with MV, an oncologist, ND, a psychologist, and LU, a sociologist.

For the present study, the existing interview transcripts were first analyzed according to thematic analysis. The categories (themes or patterns) were identified using an inductive approach on a semantic level. The data analysis was guided in a recursive process through six phases: (1) familiarizing with the data, (2) generating initial codes, (3) searching for categories, (4) reviewing categories, (5) defining and naming categories (6) reporting the categories [27].

Then for data integration, the pillar integration process was used—a systematic technique to guide data integration in four stages: (1) listing descriptive values and coded data the researchers consider important, (2) matching content that relates to initial data horizontally, (3) checking for completeness to ensure matching, and (4) pillar building by comparing and contrasting the findings and conceptualizing insights [18]. In the first stage, the listing of the descriptive values and codes was selective, including only particular data that warranted further investigation. In the second stage, similar descriptive values and codes were matched and compared by aligning, refining, and organizing categories. In the third stage, all data was cross-checked to ensure accuracy and appropriateness of the matching. In the fourth stage, insights were conceptualized by building inferences about identified patterns or themes and possible explanations [18]. Based on the descriptive values and the inductive codes quantitative and qualitative categories were derived and used for listing, matching, checking, and pillar-building to answer the research questions.

## Results

Two pillar themes emerged from comparing and contrasting the raw qualitative and quantitative data: (1) “approaching prognosis and advance care planning”, (2) “living with a life-threatening illness.” All quotes have been directly translated from German by the authors.

### Approaching prognosis and advance care planning

The qualitative interviews with patients and caregivers revealed that, in milestone conversations, prognosis and advance care planning seemed to be offered but patients’ reactions were diverse: from asking for explicit information to not wanting information, a desire for optimistic information, not wanting to be reminded about having a mortal disease, changes in processing information and readiness for information (see Table 3).

**Table 3:** Results on facilitation of prognostic awareness

Quant. data (content analysis of patient records)	Quant. categories	Pillar theme	Qual. categories	Qual. codes (qualitative semi-structured interviews)
Prognosis communication was recorded in less than 20% of the conversations (t1: 18% (*n* = 5), t2: 17% (*n* = 3) Advance care planning topics were written down 22–30% No documentation if topic was not discussed	Prognosis and advance care planning were partly documented	Approaching prognosis and advance care planning	Diverse patient reactions toward prognosis and advance care planning	“Well, I resigned to the fact that it is lethal, that it is incurable. Therefore, I came to hospice care because I would like to die in peace.” (Patient 6) “The length of the remaining period of life, one doesn´t want to know and this was respected in the conversation – the nurse and doctor in the conversation were very considerate.” (Patient 10)

*Quant.* = quantitative; *Qual.* = qualitative

Several patients reported they were offered information on prognosis but some declined:

“The length of the remaining period of life, one doesn´t want to know and this was respected in the conversation – the nurse and doctor in the conversation were very considerate.” (Patient 10)

This quote indicates that the conversation content was adapted to the patient’s wishes. Another patient explained:

“I know that I won´t regain my health 100%, but I would like that they say `you will be fine without chemotherapy, you can go to work and you will statistically live a little longer`. Do you know what I mean?” (Patient 8)

This quote indicates that, instead of realistic communication of limited prognosis including incurability, limited prognosis, and deterioration of advanced lung cancer, Patient 8 wished for an optimistic stance. Also, other patients reported not wanting to be reminded that they are terminally ill. To cope with everyday life and the situation in general, it might be easier for some patients to ignore or deny the terminal nature of the disease.

Some patients reported forgetting conversation contents and attribute that forgetfulness to a change of their cognitive functioning in these conversations:

“I think my brain turned off.” (Patient 6)

An impaired cognitive functioning might contribute to an inaccurate prognostic awareness. Several patients revealed feeling supported and not left alone through the MCA care they received. This effect was seen in one patient who reported that she agreed finally to advanced care planning after declining several times. She changed her mind because she did not want to burden her relatives with upcoming decisions.

Some patients have to deal with having advanced lung cancer while nonetheless feeling healthy and seem not to be ready for prognostic information.

“Yes, I had my scan [computed tomography] the day before yesterday. And now I just had the consultation about the results. And I am not dissatisfied and I feel fine … I don´t feel sick.” (Patient 10)

It is likely that, as long as patients do not yet perceive cancer symptoms, they are less willing to talk about prognosis and undertake advance care planning.

The interviews suggest that only a sub-group of patients appreciate discussing prognosis:

“Well, I resigned to the fact that it is lethal, that it is incurable. Therefore, I came to hospice care because I would like to die in peace.” (Patient 6)

“Yes, they told me everything that will happen. They did not sugar-coat anything about what I have to expect. And when I asked: “Oh dear, how long will the whole treatment go on?” And I liked that they openly talked to me and did not conceal anything.” (Patient 5)

Certainly, communication with patients appreciating prognostic information is easier for health care professionals than that with patients declining it.

### Living with a life-threatening illness

The interview analysis revealed several aspects that made the existential meaning of living with lung cancer clearer. It seems that living with a life-threatening illness such as lung cancer remains difficult. Patients reported struggling every day to stay positive, to live normally, and to have hope. One patient said:

“I have a positive attitude towards my illness, I don´t see it negatively.” (Patient 1)

It appears that trying to stay positive helps patients to get on with their everyday lives. Also, patients seek to live normally, meet family and friends and engage in everyday life:

“Sometimes I feel like not wishing to talk about my illness. Normal life is much more important to me.” (Patient 2)

The quote shows that it might be easier for patients to live normal lives if they are not persistently thinking or talking about their disease.

Patients’ hopes include protracting the disease, diminishing the tumor, and living longer. But these hopes are repeatedly disappointed, e.g., if the straining therapy did not succeed in the way the patient anticipated:

“Because I really thought: they give me that [the chemotherapy] and then everything [the tumor] goes away. But obviously it doesn´t work that way.” (Patient 10)

All patients, regardless of whether they were in the control or intervention group, possibly struggle with establishing their quality of life, reducing distress, and remaining in a good mood (see Table 4). This struggle might interfere with measuring these variables as outcomes for a communication intervention.

**Table 4:** Results on influencing, measuring, and perceiving a life-threatening illness

Quant. data (pragmatic rando-mized controlled trial)	Quant. categories	Pillar theme	Qual. categories	Qual. codes (qualitative semi-structured interviews)
Patients identified a median of 8.0 distress issues at t0 (MCA: median, 8.0; standard oncological care: median, 9.0) and at 3-month follow-up a median of 4.0 issues (MCA: median, 4.0; standard oncological care: median, 5.0) on the distress problem list Depression and anxiety as measured with the four-item PHQ-4 did not show differences between the treatment groups The SEIQoL quality-of-life index score showed a similar quality of life in both patient groups	There were no statistically significant differences between groups concerning distress, quality of life, or mood (anxiety/depression)	Living with a life-threatening illness	Seeking to have hope, staying positive, and having a normal life	“I have a positive attitude towards my illness, I don´t see it negatively.” (Patient 1) “Sometimes I feel like not wishing to talk about my illness. Normal life is much more important to me.” (Patient 2) “Because I really thought: they give me that (the chemotherapy) and then everything (the tumor) goes away. But obviously it doesn´t work that way.” (Patient 10)

*Quant.* = quantitative; *Qual.* = qualitative

## Discussion

Two pillar themes emerged from the data: (1) “approaching prognosis and advance care planning” and (2) “living with a life-threatening illness.” Discussions about prognosis and advance care planning have possibly been offered more often than they were documented, and an explanation for not discussing prognosis and advance care planning of some patients derived from the diverse patient reactions: not wanting information, a desire for optimistic information, not being reminded about having a mortal disease, changes in processing information, and readiness for information. In other countries, there also seems to be a subset of patients not wishing to discuss prognosis, a decline in health, or their future [28–31]. All patients seemed to struggle to preserve their quality of life, be optimistic and keep distress under control.

Healthcare professionals have to adapt to the diverse reactions of patients and their readiness to engage in the advance care planning process, and additionally to patients’ preferences, knowledge, and health literacies [32]. For clinicians confronted with patients who are clinically declining and ambivalent or renunciative towards prognostic information, the recommended communication strategy is to “name the dilemma” [9]. This approach enables clinicians to be empathic toward the patient but also addresses the disadvantages of declining information [9].

The interviews indicate that patients tried to proceed with their social life as normally as possible [33] even when their situation was deteriorating. Patients with a life-limiting illness are struggling to live in the present while facing death [34, 35]. Not fighting to stay alive or not focusing on normality seems to be morally not allowed [34]. Our findings, as those of Horne et al. (2012), suggest that patients facing death possibly only talk about what they can live with, handle, or make sense of [34]. Therefore, supporting patients in comprehensibility, meaningfulness, and manageability of lung cancer might be the key to catering for the advance care planning process [20].

From the health professionals’ perspectives, an accurate understanding of the significance of advanced disease and the goals of treatment are essential [36]. These needs can be achieved by enhancing prognostic awareness in patients. Patients declining to talk about prognosis are challenging for health care professionals for four reasons: (1) ignorance of limited prognosis hinders decision-making, notably shared decision-making [37], (2) it hinders timely referral to palliative care services and advance care planning as recommended by oncological societies [38, 39], (3) patients preferring to ignore incurability evoke in other professionals not involved in the discussions an impression of insufficient serious illness conversations, (4) the pressure of patients expressing unrealistic wishes or even demanding unrealistic treatment options is stressful for health care professionals. An ethical dilemma arises for the physician: if the physician decides to follow the principle of non-maleficence, stopping tumor treatment when harm becomes greater than benefit, it interferes with the principle of the autonomy of patients wishing for more aggressive treatment [37, 40]. Whatever the decision, it leaves physicians burdened with impairing a principle [40]. Also, patients declining to talk about prognostic information and preferring to ignore the life-limitation of the illness are possibly more often seen by physicians working in oncology [41]. Possibly these patients never, or at a very late stage, accept palliative care.

Patient-centered communication includes assessing and respecting patients’ fluctuations in prognostic awareness [9] and adapting communication on prognosis accordingly. Other research has also shown that patients prefer prognostic information disclosure on their own terms, to the degree they want [32, 42]. Appraising a patient’s degree of prognostic awareness is demanding for clinicians since it implies balancing honest prognosis communication without overwhelming the patient (with the amount of (unwanted) information). At the same time, clinicians have to ensure they do not communicate in deliberate vagueness [42]. These aspects are subtle and complex and should be documented. In healthcare, these conversations are inconsistently documented by healthcare professionals in routine patient records [43].

## Conclusion

The theoretical assumptions on communication with patients with limited prognosis may need to be reconsidered. There is an assumption that informing the patient on incurability (by using adequate communication techniques) leads to the patient linearly knowing, understanding, and acknowledging the life-limiting character of the disease. In medicine, treatment choices are not linear; they are complex, uncertain, and emotionally laden, especially in advanced cancer care [10]. Communication with patients with limited prognosis must adjust to patients’ preferences and the influence of patients’ functions, cultures, families, resources, and burden change over time [10]. Systematic explanations of serious illness conversations and patient behavior and the interrelations between concepts and definitions require further definitions and theoretical assumptions on patients’ hearing, understanding, acknowledging, and acting on a disease with a limited prognosis. In summary, further investigations are needed of the relationship between communication, information preferences, and patient-relevant outcomes.

## Strengths and limitations

Our findings contribute to the ongoing debate on how to confront patients with prognostic information. The findings on communication also reflect the ethical attitudes and values of the patients and clinicians. The integration of quantitative and qualitative methods was helpful in evaluating MCA from different perspectives and in gaining more differentiated insights on how results from MCA evaluation can be interpreted. However, mixing methods cause a higher workload. The study was performed at one outpatient department of a single academic comprehensive cancer center. The findings may not be representative of patients from other departments, with other diseases and cultural backgrounds. The raw data are from mixed quantitative and qualitative research, resulting in a reduction of information. More comprehensive information (e.g., purposive qualitative sampling, RCT blinding) would reveal more conclusive or diverging findings.

## Data Availability

Data and material are not publicly available due to the Ethical Review Board requirements and the German data protection law. The data supporting these findings are available on request from the corresponding author.

## References

[1] Ferlay J, Colombet M, Soerjomataram I, Dyba T, Randi G, Bettio M (2018). Cancer incidence and mortality patterns in Europe: estimates for 40 countries and 25 major cancers in 2018. Eur J Cancer.

[2] Allemani C, Matsuda T, Di Carlo V, Harewood R, Matz M, Nikšić M (2018). Global surveillance of trends in cancer survival 2000–14 (CONCORD-3): analysis of individual records for 37 513 025 patients diagnosed with one of 18 cancers from 322 population-based registries in 71 countries. Lancet.

[3] Arbour KC, Riely GJ (2019). Systemic therapy for locally advanced and metastatic non-small cell lung cancer: a review. JAMA.

[4] AWMF (2020) Erweiterte S3-Leitlinie Palliativmedizin für Patienten mit einer nicht heilbaren Krebserkrankung

[5] Peppercorn JM, Smith TJ, Helft PR, DeBono DJ, Berry SR, Wollins DS (2011). American Society of Clinical Oncology statement: toward individualized care for patients with advanced cancer. J Clin Oncol.

[6] Weeks JC, Catalano PJ, Cronin A, Finkelman MD, Mack JW, Keating NL (2012). Patients' expectations about effects of chemotherapy for advanced cancer. N Engl J Med.

[7] Nehls W, Gabrijel S, Kiss A, Kollmeier J, Schmalz O, Albrecht H (2013). Physician communication in a lung cancer center–does the message come across?. Pneumologie.

[8] Davison SN (2006). Facilitating advance care planning for patients with end-stage renal disease: the patient perspective. Clin J Am Soc Nephrol.

[9] Jackson VA, Jacobsen J, Greer JA, Pirl WF, Temel JS, Back AL (2013). The cultivation of prognostic awareness through the provision of early palliative care in the ambulatory setting: a communication guide. J Palliat Med.

[10] Morrison RS, Meier DE, Arnold RM (2021). What’s wrong with advance care planning?. JAMA.

[11] Bernacki RE, Block SD (2014). Communication about serious illness care goals: a review and synthesis of best practices. JAMA Intern Med.

[12] Bernacki R, Hutchings M, Vick J, Smith G, Paladino J, Lipsitz S (2015). Development of the serious illness care program: a randomised controlled trial of a palliative care communication intervention. BMJ Open.

[13] Baile WF, Buckman R, Lenzi R, Glober G, Beale EA, Kudelka AP (2000). SPIKES—a six-step protocol for delivering bad news: application to the patient with cancer. Oncologist.

[14] Grimshaw JM, Eccles MP, Lavis JN, Hill SJ, Squires JE (2012). Knowledge translation of research findings. Implement Sci.

[15] Grol R, Wensing M (2013) Effective implementation of change in healthcare: a systematic approach. In: Grol R, Wensing M, Eccles M, Davis D, editors. Improving patient care. 2nd ed: Wiley Blackwell BMJ Books; p. 40–63

[16] Creswell JW, Plano Clark VL (2018). Designing and conducting mixed methods research.

[17] Bazeley P (2009) Editorial: integrating data analyses in mixed methods research. J Mix Methods Res 3(3):203–7

[18] Johnson RE, Grove AL, Clarke A (2019) Pillar integration process: a joint display technique to integrate data in mixed methods research. J Mix Methods Res 13(3):301–20

[19] Krug K, Bossert J, Deis N, Krisam J, Villalobos M, Siegle A (2021). Effects of an interprofessional communication approach on support needs, quality of life, and mood of patients with advanced lung cancer: a randomized trial. Oncologist..

[20] Krug K, Bossert J, Stooß L, Siegle A, Villalobos M, Hagelskamp L et al (2020) Consideration of sense of coherence in a structured communication approach with stage IV lung cancer patients and their informal caregivers: a qualitative interview study. Support Care Cancer 29:2153–2159. 10.1007/s00520-020-05724-2 10.1007/s00520-020-05724-2 PMC7892692 32880008

[21] Bossert J, Wensing M, Thomas M, Villalobos M, Jung C, Siegle A (2020). Implementation of the milestones communication approach for patients with limited prognosis: evaluation of intervention fidelity. BMC Palliat Care.

[22] Tessmer G, Zaba O, Grohé C (2011). Konzept einer vorausschauenden Kommunikation in der palliativen Behandlung von Patienten mit pneumolgisch-onkologischen Erkrankung. Pneumologie.

[23] Siegle A, Villalobos M, Bossert J, Krug K, Hagelskamp L, Krisam J (2018). The Heidelberg Milestones Communication Approach (MCA) for patients with prognosis <12 months: protocol for a mixed-methods study including a randomized controlled trial. Trials.

[24] Bausewein C, Fegg M, Radbruch L, Nauck F, von Mackensen S, Borasio GD (2005). Validation and clinical application of the German version of the palliative care outcome scale. J Pain Symptom Manage.

[25] Villalobos M, Siegle A, Hagelskamp L, Handtke V, Jung C, Krug K (2019). HeiMeKOM (Heidelberger Meilenstein Kommunikation): Entwicklung einer interprofessionellen Intervention zur Verbesserung der Kommunikation bei Patient*innen mit eingeschränkter Prognose. Z Evid Fortbild Qual Gesundhwes.

[26] O'Cathain A, Murphy E, Nicholl J (2008). The quality of mixed methods studies in health services research. J Health Serv Res Policy.

[27] Braun V, Clarke V (2006). Using thematic analysis in psychology. Qual Res Psychol.

[28] Jacobsen J, Jackson VA (2009). A communication approach for oncologists: understanding patient coping and communicating about bad news, palliative care, and hospice. J Natl Compr Cancer Network: JNCCN.

[29] Hofmann JC, Wenger NS, Davis RB, Teno J, Connors Jr AF, Desbiens N (1997). Patient preferences for communication with physicians about end-of-life decisions. SUPPORT Investigators. Study to Understand Prognoses and Preference for Outcomes and Risks of Treatment. Ann Intern Med.

[30] Fried TR, Bradley EH, O'Leary J (2006). Changes in prognostic awareness among seriously ill older persons and their caregivers. J Palliat Med.

[31] Kvåle K (2007). Do cancer patients always want to talk about difficult emotions? A qualitative study of cancer inpatients communication needs. Eur J Oncol Nurs.

[32] Rietjens JAC, Sudore RL, Connolly M, van Delden JJ, Drickamer MA, Droger M (2017). Definition and recommendations for advance care planning: an international consensus supported by the European Association for Palliative Care. Lancet Oncol.

[33] van Roij J, Brom L, Youssef-El Soud M, van de Poll-Franse L, Raijmakers NJH (2019). Social consequences of advanced cancer in patients and their informal caregivers: a qualitative study. Support Care Cancer.

[34] Horne G, Seymour J, Payne S (2012). Maintaining integrity in the face of death: a grounded theory to explain the perspectives of people affected by lung cancer about the expression of wishes for end of life care. Int J Nurs Stud.

[35] Nierop-van Baalen C, Grypdonck M, van Hecke A, Verhaeghe S (2016). Hope dies last … A qualitative study into the meaning of hope for people with cancer in the palliative phase. Eur J Cancer Care.

[36] El-Jawahri A, Traeger L, Park ER, Greer JA, Pirl WF, Lennes IT (2014). Associations among prognostic understanding, quality of life, and mood in patients with advanced cancer. Cancer.

[37] Vos MS, Putter H, van Houwelingen HC, de Haes HC (2011). Denial and social and emotional outcomes in lung cancer patients: the protective effect of denial. Lung Cancer.

[38] Ferrell BR, Temel JS, Temin S, Alesi ER, Balboni TA, Basch EM (2017). Integration of palliative care into standard oncology care. Am Soc Clin Oncol Clin Pract Guidel Update.

[39] AWMF, DKG, DKH. S3-Leitlinie Prävention, Diagnostik, Therapie und Nachsorge des Lungenkarzinoms 2018 [Available from: http://leitlinienprogramm-onkologie.de/Lungenkarzinom.98.0.html. Accessed 20 June 2022

[40] Beauchamp T, Childress J (2019). Principles of biomedical ethics: marking its fortieth anniversary. Am J Bioeth: AJOB.

[41] Pfeil TA, Laryionava K, Reiter-Theil S, Hiddemann W, Winkler EC (2015). What keeps oncologists from addressing palliative care early on with incurable cancer patients? An active stance seems key. Oncologist.

[42] Innes S, Payne S (2009). Advanced cancer patients' prognostic information preferences: a review. Palliat Med.

[43] Heyland DK, Ilan R, Jiang X, You JJ, Dodek P (2016). The prevalence of medical error related to end-of-life communication in Canadian hospitals: results of a multicentre observational study. BMJ Qual Saf.

